# Strong Genetic Influences on the Stability of Autistic Traits in Childhood

**DOI:** 10.1016/j.jaac.2013.11.001

**Published:** 2014-02

**Authors:** Karla Holmboe, Fruhling V. Rijsdijk, Victoria Hallett, Francesca Happé, Robert Plomin, Angelica Ronald

**Affiliations:** a Department of Psychology at the the University of Essex; b Medical Research Council (MRC) Social, Genetic and Developmental Psychiatry Centre, Institute of Psychiatry, King's College London; c Department of Psychological Sciences, Birkbeck, University of London

**Keywords:** autism spectrum disorder, autistic traits, behavior genetics, longitudinal

## Abstract

**Objective:**

Disorders on the autism spectrum, as well as autistic traits in the general population, have been found to be both highly stable across age and highly heritable at individual ages. However, little is known about the overlap in genetic and environmental influences on autistic traits across age and the contribution of such influences to trait stability itself. The present study investigated these questions in a general population sample of twins.

**Method:**

More than 6,000 twin pairs were rated on an established scale of autistic traits by their parents at 8, 9, and 12 years of age and by their teachers at 9 and 12 years of age. Data were analyzed using structural equation modeling.

**Results:**

The results indicated that, consistently across raters, not only were autistic traits stable, and moderately to highly heritable at individual ages, but there was also a high degree of overlap in genetic influences across age. Furthermore, autistic trait stability could largely be accounted for by genetic factors, with the environment unique to each twin playing a minor role. The environment shared by twins had virtually no effect on the longitudinal stability in autistic traits.

**Conclusions:**

Autistic traits are highly stable across middle childhood. and this stability is caused primarily by genetic factors.

Autism spectrum disorder (ASD) is a neurodevelopmental disorder characterized by impairments in social interaction (SIs) and communication (CIs), as well as restricted and repetitive behaviors and interests (RRBIs). The spectrum ranges from severe (typically accompanied by intellectual impairment and lack of language) to relatively high functioning. With the introduction of *DSM-5*, several disorders previously labeled as pervasive developmental disorders have been merged into a single ASD diagnosis. Furthermore, the 2 symptom domains of SIs and CIs have been combined to form a single social communication impairment domain.^1^ Autistic traits show quantitative variation in the general population.^2^, ^3^, ^4^, ^5^, ^6^, ^7^

ASD is both highly heritable and highly stable over time. Longitudinal studies have found that individuals diagnosed with ASD rarely move off the spectrum (but see Fein *et al.*^8^), although improvement in symptoms is often observed between childhood and adolescence/adulthood (for review, see Matson and Horovitz^9^). Similarly, high stability has been found when autistic traits are measured in the general population.^10^, ^11^, ^12^

The heritability of ASD has been established in family and twin studies (the twin method is detailed fully in Supplement 1^13^). Twin studies in the United Kingdom, United States, Sweden, and Japan have established that the concordance rate for ASD in monozygotic (MZ) twins is much higher (36–96%) than in dizygotic (DZ) twins (0–36%), suggesting substantial genetic influences (73–93%).^14^ Likewise, twin studies of autistic traits in community and population-based samples in middle childhood and adolescence have found evidence for moderate to strong genetic effects (generally ranging between 50% and 90%), with the remaining variance primarily explained by non-shared environmental factors.^14^, ^15^, ^16^ There is now evidence that the 3 symptom domains of SIs, CIs, and RRBIs, although all highly heritable, are influenced by only partially overlapping genetic factors.^7^, ^17^, ^18^, ^19^, ^20^ The specific genetic mechanisms underlying ASD have also been investigated extensively in recent molecular genetic research (for review, see Abrahams and Geschwind^21^).

Despite extensive research into the causal influences on autistic traits at individual ages and the finding of high stability in these traits, very few studies have investigated the causal influences on autistic trait stability itself. Likewise, little is known about whether causal influences overlap across age. Knowing this is important for understanding the developmental course of autistic traits. Constantino *et al.* followed up a small sample of 95 male twin pairs (aged 3–18 years) from the general population for 5 years and found that change in parent-reported autistic traits across the period could be accounted for largely by genetic factors (73%).^10^ These authors also found some limited evidence that genetic influences in childhood overlapped only partially with those in adolescence.

In the present study, our aim was to establish the causal influences on the longitudinal development of autistic traits across middle childhood. We were also interested in investigating sex differences in these influences, given evidence that more boys than girls are diagnosed with ASD,^22^, ^23^ and the finding of sex differences in large cross-sectional twin studies of autistic traits.^5^, ^7^, ^17^ Furthermore, different raters provide information on a child's autistic-like behaviors in different contexts,^24^ and it is therefore important to test whether the causal model of stability in autistic traits is the same across raters. Finally, little is known about the longitudinal development of the three domains of autistic symptoms. The partial independence of SIs, CIs, and RRBIs has been established previously.^7^, ^17^, ^18^, ^20^ However, it remains unresolved whether similar causal effects operate on these 3 dimensions across development.

To address these questions, a population-based sample of more than 6,000 twin pairs was assessed. Autistic traits were investigated primarily as an overall trait, but the 3 dimensions of SIs, CIs, and RRBIs were also explored. Male and female twins were assessed on the same measure of autistic traits by teachers at ages 9 and 12 years and by parents at ages 8, 9, and 12 years, allowing investigation of sex differences and rater differences in causal effects.

## Method

### Participants

Participants came from the Twins Early Development Study (TEDS), a population-based longitudinal study of all twins born in England and Wales from 1994 to 1996. The present paper is based on data collected from parents when the twins were approximately 8 (mean ± SD = 7.89 ± 0.53), 9 (9.01 ± 0.29), and 12 (11.28 ± 0.70) years old, and from teachers when the twins were approximately 9 (9.04 ± 0.29) and 12 (11.54 ± 0.66) years old. Zygosity in same-sex twins was established using a parent-rated twin similarity questionnaire^25^ (60.5% of twins) and DNA genotyping (39.5% of twins). Twin pairs were excluded from the analyses if severe pre- or postnatal complications were reported or if either twin had a severe medical condition. To retain the full range of variability in autistic traits, children who had suspected or confirmed ASD (n = 238) based on the Development and Well-Being Assessment (DAWBA)^26^ were included in the analyses when data were available. After exclusions, parent-reported data on both twins were available from 6,280 families at age 8 years, 3,126 families at age 9 years (the 9-year sample was a subsample of families with twins born between January 1994 and August 1995), and 5,339 families at age 12 years. Teacher data were available from 2,663 families at age 9 and 4,405 families at age 12. The sample at each of these ages was representative of the U.K. population.^20^, ^24^, ^27^ Further details on the recruitment and study protocol are reported elsewhere.^28^, ^29^

### Measures

Autistic traits were assessed using the Childhood Autism Spectrum Test (CAST, formerly the Childhood Asperger Syndrome Test).^30^ The CAST was developed as a screening questionnaire for use in non-clinical settings. The published measure consists of 37 items, 31 of which relate to autistic traits (raters are asked to rate the child on specific behaviors associated with autistic traits), the remaining 6 being control questions on general development. At age 12 years, 1 item (regarding pretend play) was removed for not being age appropriate. Each question was rated “Yes” (1)/“No” (0) at ages 8 and 12, and as “Not true” (0), “Somewhat true” (1), or “Certainly true” (2) at age 9. Questionnaires with half or more of the items rated were included in the analyses. Only between 0.19% and 2.38% of questionnaires were excluded based on this criterion at any individual age. CAST subscale scores were calculated using the method described by Ronald *et al.*^7^, ^31^

The CAST has good internal consistency: in the present study Cronbach's α ranged from 0.71 to 0.81, depending on age and rater. It has high sensitivity and specificity for ASD (both > 95%) using a cut-point of ≥ 15,^32^ as well as high test–retest reliability.^33^ Recently, the CAST has been used successfully in epidemiological research to screen for previously undetected cases of ASD.^23^

At ages 8 and 12 years, the full CAST was used. At age 9, the 20-item abbreviated version of the CAST was used.^24^ Between ages 8 and 9, the full and abbreviated versions of the CAST correlated at 0.63,^21^ which for the same measure would be considered acceptable test–retest reliability across a 1-year interval (see, e.g., test–retest reliability of the Strengths and Difficulties Questionnaire^34^), supporting the abbreviated version as an equivalent but shorter version of the CAST. Correlations between teacher and parent ratings were modest at age 9 (*r* = .32, *p* < .001) and 12 (*r* = .25, *p* < .001). Similar levels of low to moderate agreement between teacher and parent ratings have been reported in other community-based studies of behavior problems and autistic traits using a variety of established measures (reviewed by Ronald *et al.*^24^). For this reason, the parent and teacher data were analyzed separately.

### Data Analyses

Information on the general principles of the twin design and model fitting can be found in Supplement 1.^13^ Structural equation modeling (SEM) analyses of the raw twin data were carried out using the software program *Mx*.^35^ Before modeling, CAST scores were first corrected for mean effects of age and sex using regression in SPSS Statistics 19 (IBM SPSS, Chicago, IL), and then log transformed to remove skew. A saturated model was then specified in *Mx*. An unconstrained saturated model accounts for all of the variance and covariance in the observed data without any model assumptions imposed, and will therefore always fit the data perfectly. However, to meet the theoretical assumptions for modeling the variance components (additive genetic [A], shared environmental [C], non-additive/dominant genetic [D], and non-shared environmental [E]) and their correlations (rA, rC, rD, and rE), the saturated model was constrained so that, when twin 1 and twin 2 were the same sex, they had the same mean, SD, and correlations. Mean, SD, and phenotypic correlations were also constrained to be the same for males and females separately (e.g., all male individuals, whether part of an MZ, DZ same-sex, or DZ opposite-sex pair, were assumed to have the same mean, SD, and phenotypic correlation). However, males and females were allowed to differ in their mean, SD, and twin correlations. Using this constrained saturated model, the phenotypic longitudinal correlations, the twin correlations at each age, and the cross-twin cross-age correlations were estimated.

A multivariate quantitative sex-limitation model incorporating equal correlation matrices for males and females was used for both teacher and parent data (see “Scalar sex limitation: correlation approach” in Neale *et al.*^36^; note that these investigators use the term “scalar sex limitation” to denote a model involving quantitative sex differences). Using this model, we were able to include data from DZ opposite-sex pairs under the constraint that variance component correlations were specified to be the same for males and females. The inclusion of DZ opposite-sex data provided considerable extra power and therefore more accurate parameter estimates. Following specification of the parent and teacher models, the causal influence of parameters A, C/D, and E on the longitudinal stability in autistic traits was examined using bivariate estimates (for details, see Supplement 1^13^).

## Results

### Descriptive Statistics and Phenotypic Longitudinal Correlations

Means and SDs for each sex × zygosity group can be found in Supplement 2.^13^ Table S1^13^ presents descriptive statistics for males and females at each age. Females scored significantly lower than males on the CAST and had significantly smaller SDs at all ages (*p* < .001). This is consistent with previous research on both diagnosed ASD and autistic traits.^6^, ^22^, ^23^ Also consistent with previous findings,^10^, ^11^ there was a slight but significant (*t*_4,400_ = 6.42, *p* < .001) decrease in parent-rated CAST scores in males between 8 and 12 years of age (comparison of mean scores at age 9 with mean scores at other ages was not possible because scores were rated on a 0–2 scale instead of a 0–1 scale). However, in the present study this decrease was also seen in females (*t*_4,952_ = 4.43, *p* < .001).

Table 1 presents the phenotypic longitudinal correlations. Parent ratings of autistic traits showed moderately high stability with correlations ranging between 0.59 and 0.67. Teacher ratings showed more modest stability with a correlation of 0.35 for males and 0.26 for females between ages 9 and 12. All longitudinal correlations were significant (*p* < .001). The longitudinal correlations were slightly lower in females than in males (*p* < .01).

**Table 1:** Longitudinal Correlations for Autistic Traits Assessed by the Childhood Autism Spectrum Test

Parent Ratings	Males	Females
8 → 9 years	0.67 (0.65–0.69)	0.63 (0.61–0.65)
8 → 12 years	0.67 (0.66–0.69)	0.60 (0.58–0.62)
9 → 12 years	0.66 (0.64–0.69)	0.59 (0.56–0.61)

Teacher ratings	Males	Females
9 → 12 years	0.35 (0.30–0.40)	0.26 (0.21–0.31)

Note: Data in parentheses are 95% CI.

### Twin Correlations

Table 2 contains the within-age and cross-age twin correlations (for details on the interpretation of twin correlations, see Supplement 1^13^). The within-age (univariate) twin correlations can be seen on the diagonal for each sex × zygosity group (marked in boldface). For all age groups, both sexes, and both types of rater, MZ correlations were considerably higher than DZ correlations, indicating the presence of genetic influences on autistic traits. Modest differences between male and female same-sex twin correlations suggested the existence of quantitative sex differences. DZ opposite-sex correlations were generally not substantially lower than DZ same-sex correlations, which would have indicated qualitative sex differences. The presence of modest dominant genetic or shared environmental influences was not consistent across ages, sexes and raters, but was indicated in some cases. Most noticeable was the suggestion of modest dominant genetic effects for males at ages 8 and 12 years (but not at age 9) in the parent data. In contrast, a small effect of the shared environment was indicated in the teacher data and for females generally. In none of the groups did the MZ twin correlation reach 1, indicating that non-shared environmental influences, including measurement error, played a role.

**Table 2:** Twin Correlations for the Childhood Autism Spectrum Test at Ages 8, 9, and 12 Years

Parent Ratings, y (n)	8 Years	9 Years	12 Years	Teacher Ratings, y (n)	9 Years	12 Years
MZ males						
8 (1,067)	**0.79 (0.77–0.81)**					
9 (543)	0.56 (0.53–0.59)	**0.83 (0.81–0.85)**		9 (456)	**0.69 (0.64–0.73)**	
12 (921)	0.59 (0.56–0.61)	0.57 (0.54–0.60)	**0.79 (0.77–0.81)**	12 (761)	0.35 (0.29–0.41)	**0.59 (0.54–0.63)**
DZ males						
8 (1,042)	**0.26 (0.21–0.32)**					
9 (515)	0.23 (0.17–0.28)	**0.48 (0.41–0.54)**		9 (431)	**0.43 (0.35–0.50)**	
12 (863)	0.17 (0.12–0.22)	0.24 (0.18–0.30)	**0.29 (0.23–0.35)**	12 (706)	0.19 (0.10–0.27)	**0.31 (0.24–0.38)**
MZ females						
8 (1,189)	**0.80 (0.78–0.82)**					
9 (674)	0.57 (0.55–0.60)	**0.88 (0.86–0.89)**		9 (544)	**0.68 (0.63–0.72)**	
12 (1,121)	0.53 (0.51–0.56)	0.53 (0.50–0.55)	**0.76 (0.74–0.78)**	12 (914)	0.24 (0.18–0.30)	**0.51 (0.46–0.55)**
DZ females						
8 (1,051)	**0.43 (0.38–0.47)**					
9 (559)	0.30 (0.25–0.35)	**0.55 (0.49–0.59)**		9 (476)	**0.31 (0.23–0.38)**	
12 (983)	0.27 (0.23–0.31)	0.29 (0.24–0.33)	**0.43 (0.38–0.48)**	12 (831)	0.13 (0.06–0.20)	**0.34 (0.28–0.40)**
DZ opposite sex						
8 (2,061)	**0.38 (0.34–0.41)**					
9 (1,010)	0.33 (0.30–0.36)	**0.53 (0.48–0.56)**		9 (874)	**0.36 (0.30–0.42)**	
12 (1,771)	0.27 (0.23–0.30)	0.30 (0.26–0.34)	**0.37 (0.33–0.41)**	12 (1,453)	0.10 (0.04–0.16)	**0.20 (0.15–0.25)**

Note: Data in parentheses are 95% CI. The diagonal values (in boldface type) are the within-age correlations between twin 1 and twin 2, whereas the off-diagonal values indicate the cross-twin cross-age correlations. DZ = dizygotic; MZ = monozygotic; y = years.

MZ cross-twin cross-age correlations were substantially higher than the equivalent DZ correlations (seen in the off-diagonal values in Table 2), suggesting that additive genetic influences have an impact on the longitudinal stability of autistic traits. In the parent data, the cross-twin cross-age correlations for MZ twins appeared smaller than the within-individual longitudinal (phenotypic) correlations, indicating that non-shared environmental factors may play a small role in the stability of autistic traits; however, in the teacher data, the MZ cross-twin cross-age correlation was virtually the same as the phenotypic correlation. The presence of dominant genetic or shared environmental influences on the longitudinal stability of autistic traits was not consistent between the different ages.

### Models and Parameter Estimates

As detailed above, the twin correlations from the teacher scores suggested that an ACE model would fit the data best. The parent data were more complex in that the correlations for males at ages 8 and 12 suggested an ADE model, whereas the 9-year data for males suggested an ACE model. In females, the parent data at all ages indicated that an ACE model would fit best. Therefore, a hybrid model was implemented to account for the parent data, specifying ADE at ages 8 and 12 and ACE at age 9 for males, and ACE for females at all ages. MZ and DZ variances were not significantly different (*p* > .05), indicating minimal sibling interaction.

The multivariate twin models described in the Data Analyses section were implemented in *Mx* and are presented in Table 3. The parameter estimates for parent-rated data indicated a high contribution of additive genetic factors (A) to individual differences in autistic traits at each age for both sexes (68–76%). Furthermore, the overlap in these additive genetic influences across ages (rA) was substantial: all genetic correlations were > 0.70. There was a small, but significant, contribution of shared environmental influences to parent-rated autistic traits at all ages for girls and at age 9 for boys (7–17%). The overlap in these influences across age (rC) for girls was minimal. In boys, there was a small significant contribution of dominant genetic influences to parent-rated autistic traits at ages 8 and 12; the overlap in these influences was very high (rD = 1). There was a consistent contribution of non-shared environmental influences in both sexes at all ages to parent-rated autistic traits (13–24%). The overlap in these influences across age (rE) was modest (0.37–0.47).

**Table 3:** Fit Statistics and Parameter Estimates (95% CI in Parentheses) From the Multivariate Twin Models of Parent-Rated and Teacher-Rated Childhood Autism Spectrum Test Scores

Model	−2LL	*df*	Parameters	Δ χ^2^ (*df*)	*p*	AIC	
Parent Ratings							
Saturated^a^	85045.83	30700	48				
Multivariate model^b^	85127.18	30714	34	81.40 (14)	<.01	53.40	
Parameter estimates							
Males	A	D/C^c^	E		rA	rD^d^	rE
8 years	0.71 (0.64–0.74)	0.09 (0.03–0.12)	0.20 (0.19–0.22)	8–9 years	0.78 (0.75–0.80)		0.47 (0.42–0.47)
9 years	0.76 (0.71–0.80)	0.09 (0.05–0.11)	0.15 (0.13–0.17)	8–12 years	0.72 (0.69–0.73)	1.00 (0.77–1.00)	0.37 (0.33–0.37)
12 years	0.74 (0.68–0.78)	0.06 (0.01–0.12)	0.20 (0.18–0.24)	9–12 years	0.78 (0.74–0.89)		0.42 (0.37–0.46)
Females	A	C	E		rA	rC	rE
8 years	0.71 (0.64–0.79)	0.08 (0.01–0.08)	0.21 (0.19–0.23)	8–9 years	0.78 (0.75–0.80)	0.05 (0.02–0.05)	0.47 (0.42–0.47)
9 years	0.70 (0.62–0.70)	0.17 (0.10–0.20)	0.13 (0.12–0.13)	8–12 years	0.72 (0.69–0.80)	*0.22 (−0.67 to 0.22)*	*0.37 (−0.73 to 0.37)*
12 years	0.68 (0.61–0.68)	0.07 (0.01–0.07)	0.24 (0.22–0.27)	9–12 years	0.78 (0.74–0.82)	*−0.19 (−0.27 to 0.20)*	0.42 (0.37–0.43)
Teacher Ratings							
Saturated^a^	46241.41	14934	25				
Multivariate model^b^	46243.054	14940	19	1.64 (6)	NS	-10.36	
Parameter estimates							
Males	A	C	E		rA	rC^e^	rE
9 years	0.56 (0.43–0.70)	0.13 (0.01–0.25)	0.31 (0.27–0.34)	9–12 years	0.58 (0.44–0.71)		*0.03 (−0.05 to 0.05)*
12 years	0.59 (0.50–0.59)	*0.00 (0.00–0.07)*	0.41 (0.37–0.45)				
Females	A	C	E		rA	rC^e^	rE
9 years	0.65 (0.56–0.70)	*0.02 (0.00–0.09)*	0.33 (0.29–0.37)	9–12 years	0.58 (0.44–0.71)		*0.03 (−0.05 to 0.07)*
12 years	0.35 (0.22–0.35)	0.16 (0.03–0.27)	0.49 (0.45–0.52)				

Note: Parameter estimates in italics were not significant (CI overlapped with 0). A = additive genetic influences; AIC = Akaike's Information Criterion; C = shared environment influences; D = dominant genetic influences; df = degrees of freedom; E = non-shared environment influences; p = probability; rA = additive genetic correlation; rC = shared environment correlation; rD = dominant genetic correlation; rE = non-shared environment correlation; −2LL = log likelihood fit statistic; Δ χ^2^ = difference χ^2^ between constrained saturated and multivariate model.

a Constrained saturated model.

b Multivariate quantitative sex-limitation model incorporating equal correlation matrices for males and females.^36^

c Dominant genetic influences (D) at 8 and 12 years, shared environment influences (C) at 9 years.

d Dominant genetic correlation (rD) is possible only between 8 and 12 years because C was estimated at 9 years.

e Shared environment correlation (rC) between 9 and 12 years was not possible because C was significant only at 1 of the ages (CI overlapped with 0 at age 12 years for males and age 9 years for females).

The teacher data showed a more modest contribution of additive genetic factors to autistic traits at individual ages (35–65%). The overlap in these influences between 9 and 12 years was considerable (rA = 0.58). The effect of shared environmental factors was small and inconsistent, only reaching significance at 9 years in males and 12 years in females on teacher-rated autistic traits. Because the effect of the shared environment was only present at 1 age for each sex, a shared environment correlation could not be estimated. Finally, the teacher data suggested a moderate effect of non-shared environmental factors (31–49%) at individual ages. However, these factors did not overlap across age (rE close to 0), indicating that different non-shared environmental influences were at play at each age.

### Contribution of Causal Influences to the Longitudinal Stability in Autistic Traits

**Figure 1:**
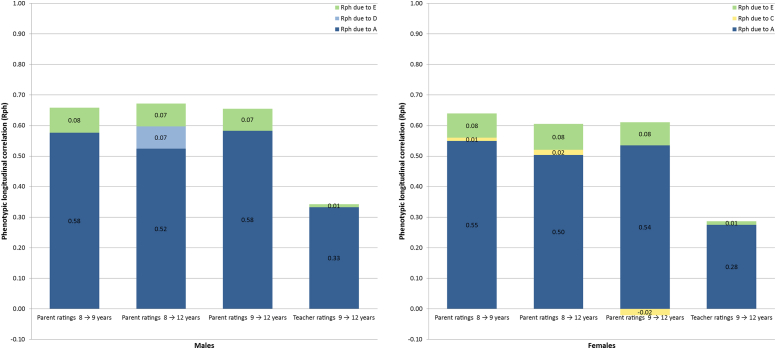
Contribution of causal influences to the longitudinal stability in autistic traits. Note: The height of each bar indicates the phenotypic correlation between ages, whereas the space between the top of each bar and 1.00 indicates the remaining variance, that is, variance not shared between ages. The differently shaded segments indicate the size of the contribution of each variance component to the longitudinal correlation. Rph = phenotypic longitudinal correlation; A = additive genetic influences; C = shared environment influences; D = dominant genetic influences; E = non-shared environment influences.

Figure 1 presents, separately for boys and girls, the contribution of each variance component to the longitudinal stability in autistic traits according to the multivariate twin models. Between all ages, and for both raters and sexes, additive genetic factors accounted for most of the longitudinal stability in autistic traits. In the parent data, there was also a small effect of the non-shared environment on longitudinal stability, as well as a small contribution of dominant genetic effects to the longitudinal stability between 8 and 12 years in boys. The effect of the shared environment on longitudinal stability in autistic traits was non-significant in boys and minimal in girls. In the teacher data, the longitudinal stability in autistic traits could be accounted for almost entirely by additive genetic factors.

### Subscale Analyses

The same set of analyses as described for the total CAST were carried out on each of the 3 CAST subscales (SIs, CIs, and RRBIs). For simplicity, and because the teacher data provided a minimum estimate of longitudinal stability (see Discussion), subscale analyses were carried out only on the parent data. The results of these analyses are presented in the supplementary material to this article:^13^ Table S2 presents the longitudinal phenotypic correlations for each subscale; Tables S3–S5 present the twin correlations, and Tables S6–S8 present the modeling results.

The subscale results were broadly consistent with the findings from the total CAST scale. Longitudinal phenotypic correlations were slightly lower for the subscales (*r* = 0.33–0.62), particularly for SIs and RRBIs. Additive genetic correlations tended to be moderate to high (rAs > 0.5), the exception being the SIs subscale, which demonstrated only modest additive genetic correlations (rAs = 0.25–0.31). Dominant genetic correlations, where present, were very high (rDs ≥ 0.85). Non-shared environment correlations were modest for all subscales (rEs = 0.27–0.39). Because of the inconsistent presence of the C component, it was not possible to estimate a shared environment correlation in the subscale models.

Figures S1 to S3^13^ present the contribution of each variance component to the longitudinal stability in SIs, CIs, and RRBIs respectively. For all 3 subscales, genetic factors accounted for most of the longitudinal stability, although subscales differed in whether these genetic factors were primarily additive (CIs) or non-additive (SIs). The shared environment played a negligible role in longitudinal stability. Finally, in accordance with findings from the total scale, non-shared environmental influences played a modest but significant role in longitudinal stability.

## Discussion

The present study is the first large-scale, population-based twin study of genetic and environmental influences on autistic traits longitudinally. Consistent with previous evidence,^10^, ^11^, ^12^ CAST scores were significantly and substantially correlated between 8 and 12 years (Table 1). The parent-rated scores showed the highest longitudinal correlations (*r* = 0.59–0.67). The longitudinal correlations in teacher-rated scores were lower (*r* = 0.26–0.35). A plausible explanation for the lower stability in teacher ratings is the fact that twins were usually rated by different teachers (but the same parent) at each age. The teacher data therefore provide a minimum estimate of stability. Nevertheless, it is noteworthy that even with different raters for the same individuals, autistic traits showed a significant degree of stability across age.

Also replicating previous findings,^14^, ^20^ we found that autistic traits as a whole were moderately to highly heritable at each age. Approximately 70% of the variance could be accounted for by additive genetic factors when children were rated by their parents. The equivalent figures for the teacher data were around 55% to 65%, the only exception being the relatively modest contribution of additive genetic effects (35%) to teacher-rated autistic traits in females at age 12. The smaller contribution of additive genetic influences to variation in teacher-rated autistic traits was matched by somewhat higher contributions of the non-shared environment (31–49%). It is worth noting that measurement error (included in the E parameter) is likely to have been larger in the teacher data because twins were often rated by different teachers, with different levels of knowledge about them. An increase in E puts an upper cap on the A parameter, perhaps explaining some of the discrepancy in heritability estimates between teacher-rated and parent-rated scores.

The present study found that the overlap in genetic influences across middle childhood is substantial. In the parent-rated data on total autistic traits, the additive genetic correlation between ages was consistently higher than 0.70, indicating that most of the additive genetic variance was shared across age. A similar pattern emerged in the teacher data: although it was slightly lower than the parent estimates, the additive genetic correlation of 0.58 between 9 and 12 years suggested considerable overlap in genetic influences across age. Furthermore, the bivariate heritability and bivariate environmental estimates revealed that longitudinal stability in autistic traits was largely due to genetic effects (Figure 1). The subscale analyses of SIs, CIs, and RRBIs broadly replicated these results. Again, longitudinal stability in each trait could be accounted for primarily by genetic effects.

The findings of the present study have important implications for understanding ASD. They suggest that autistic traits are not just highly stable across middle childhood, but that this stability is caused largely by genetic influences in both boys and girls. This implies that, if these findings generalize to the clinical extreme, stability is a biological characteristic of autistic symptoms, as opposed to, for example, being due to shared environmental factors. However, the results do not mean that all variance in the stability of autistic traits is caused by genetic influences, nor do they suggest that autistic traits cannot be changed. Even though the correlation in genetic influences was generally around 0.60 to 0.80 between ages, in no instances did confidence intervals reach 1, suggesting the presence of age-specific genetic influences as well as genetic influences common across age. Notably, the environment shared by the twins, often thought of as the family environment, had virtually no influence on the stability of autistic traits. This was despite some age-specific influences of the shared environment. In contrast, the environment unique to each twin, such as being in different classes at school or receiving differential parental treatment, did seem to play a modest role in the stability of autistic traits.

Overall, the findings suggest that the development of autistic traits in middle childhood is characterized mainly by a set of stable genetic influences, but that, in addition to these influences, new genetic influences as well as modest-to-moderate environmental influences emerge at each age. The specific nature of both these stable and temporary causal influences on autistic traits is an important topic for future research.

The strengths of the present study include its large sample size and the corroboration of findings across raters. However, the study also has limitations. One issue relates to the findings at 9 years of age. An abbreviated version of the CAST was used at this age, and items were rated on a 0–2 scale instead of a 0–1 scale. This makes comparison of mean scores difficult across ages. Furthermore, the analyses at this age had less power than at other ages. This might explain why for boys a dominant genetic effect at 8 and 12 years was replaced by a shared environment effect at age 9. We also cannot rule out that this could partly be due to small differences between the full and abbreviated CAST.

There are also more general limitations of the present study. Despite evidence for a continuous distribution of autistic traits in the general population with no clear diagnostic cut-point,^37^ the findings need replication in a clinical ASD sample. The twin design involves important assumptions that need to be met to ensure valid findings, such as the equal environments assumption and the absence of assortative mating (for discussion, see Rijsdijk and Sham^38^ and Plomin *et al.*^39^). Furthermore, gene × environment interaction (G × E) cannot be estimated in the classical twin design, where any such interaction is included in the A parameter. Finally, the present study relied on parent and teacher ratings. Even though it is a strength of the study to have multiple raters, results would benefit from being corroborated using direct observation. Future research should endeavor to explore the molecular genetic substrates of the stability in autistic traits, potential G × E interaction, and the causes of stability across the lifespan, which were beyond the scope of this study.
